# Supplementary material for: Molecular insights into Spindlin1-HBx interplay and its impact on HBV transcription from cccDNA minichromosome
Source: Nat Commun. 2023 Aug 3;14:4663. doi: 10.1038/s41467-023-40225-w (PMC10400593; doi:10.1038/s41467-023-40225-w)
Supplement: Supplementary file 1 — Supplementary Information [file 41467_2023_40225_MOESM1_ESM.pdf]

# Supplementary Information

# Molecular insights into Spindlin1-HBx interplay and its impact on HBV transcription from cccDNA minichromosome

Wei Liu[1,2], Qiyan Yao[3,4], Xiaonan Su[1,2], Yafang Deng[1], Mo Yang[5,7], Bo Peng[3], Fan Zhao[1,2], Chao Du[1,2], Xiulan Zhang[1], Jinsong Zhu[5,8], Daliang Wang[1 *], Wenhui Li[3,6 *] and Haitao Li[1,2 *]

[1]State Key Laboratory of Molecular Oncology, MOE Key Laboratory of Protein Sciences, Beijing Frontier Research Center for Biological Structure, SXMU-Tsinghua Collaborative Innovation Center for Frontier Medicine, School of Medicine, Tsinghua University, Beijing 100084, China

[2]Tsinghua-Peking Center for Life Sciences, Beijing 100084, China

[3]National Institute of Biological Sciences, Beijing 102206, China

[4]Graduate School of Peking Union Medical College, Chinese Academy of Medical Sciences, Beijing 100730, China

[5]National Center for Nanoscience and Technology, Beijing 100190, China.

[6]Tsinghua Institute of Multidisciplinary Biomedical Research, Tsinghua University, Beijing 100084, China

[7]Present address: Chemical Biology Laboratory, National Cancer Institute, 1050 Boyles Str., Frederick, MD 21702, USA

[8]Present address: Suzhou Puxin Life Science Technology, Ltd, Suzhou 215124, China

These authors contributed equally: Wei Liu, Qiyan Yao

*Correspondence: wangdaliang@tsinghua.edu.cn; liwenhui@nibs.ac.cn; lht@tsinghua.edu.cn.

**This Supplementary Information file contains:**

**Supplementary Figures 1-5**

**Supplementary Tables 1-4**

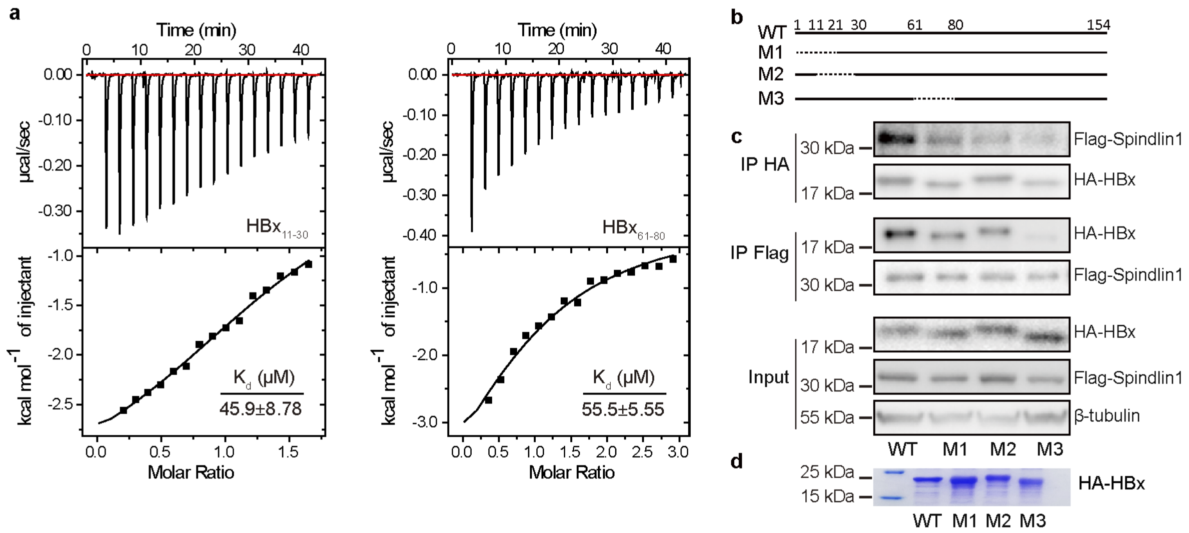


**Supplementary Figure 1. Spindlin1 interacts with HBx.** (a) ITC fitting curves of $HBx_{11-30}$ and $HBx_{61-80}$ peptides titrated to human $Spindlin1_{50–262}$ protein. (b) Determination of the region of HBx interacting with Spindlin1. Schematic representation of the full-length WT and mutant HBx protein. (c) HEK 293 T cells were co-transfected with Flag-Spindlin1 and HA-tagged WT or mutant HBx containing a substitution of 20 consecutive amino acids by alanine (M1=$HBx_{2-21}$ mutant, M2=$HBx_{11-30}$ mutant and M3=$HBx_{61-80}$ mutant). Cellular extracts were immunoprecipitated with anti-Flag M2 agarose beads and anti-HA antibodies and analyzed by the indicated antibodies using WB. β-tubulin was measured and analyzed as an input control. n = 3 independent experiments. (d) SDS-PAGE gel showing the band positions of the four purified proteins: WT, M1, M2 and M3. n = 2 independent experiments. Source data are provided as a Source Data file.

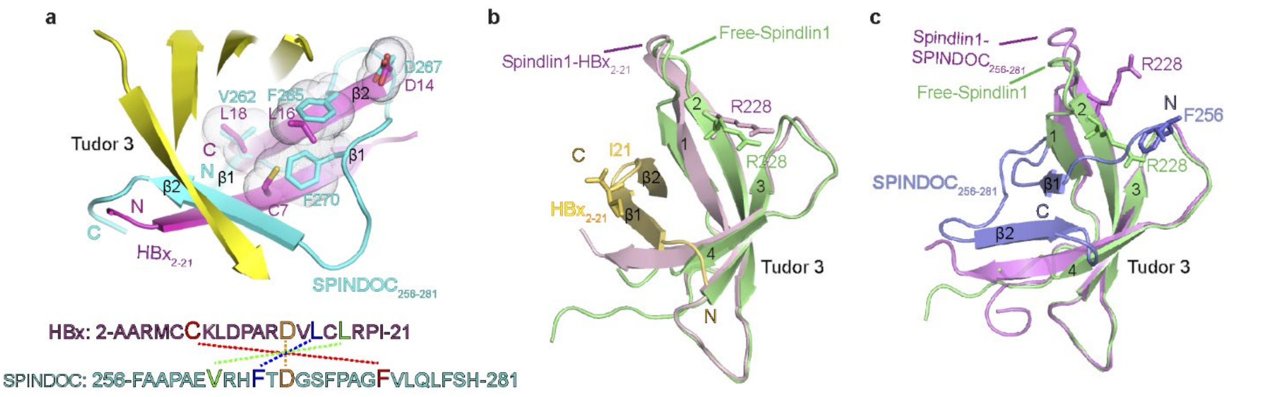


**Supplementary Figure 2. Structural comparison of Spindlin1-HBx$_{2-21}$ complex with Spindlin1-SPINDOC$_{256-281}$ complex.** (a) Both HBx$_{2-21}$ (light magenta) and SPINDOC$_{256-281}$ (cyan) complete the β-barrel fold of Spindlin1 Tudor 3 (yellow) with similar hydrophobic core formation and β-sheet formation. (b-c) Structural alignment of free Spindlin1 Tudor 3 (green) (PDB ID 2NS2) with Spindlin1-HBx$_{2-21}$ complex (b, pink-yellow) or Spindlin1-SPINDOC$_{256-281}$ (c, violet-slate, PDB ID 7E9M).

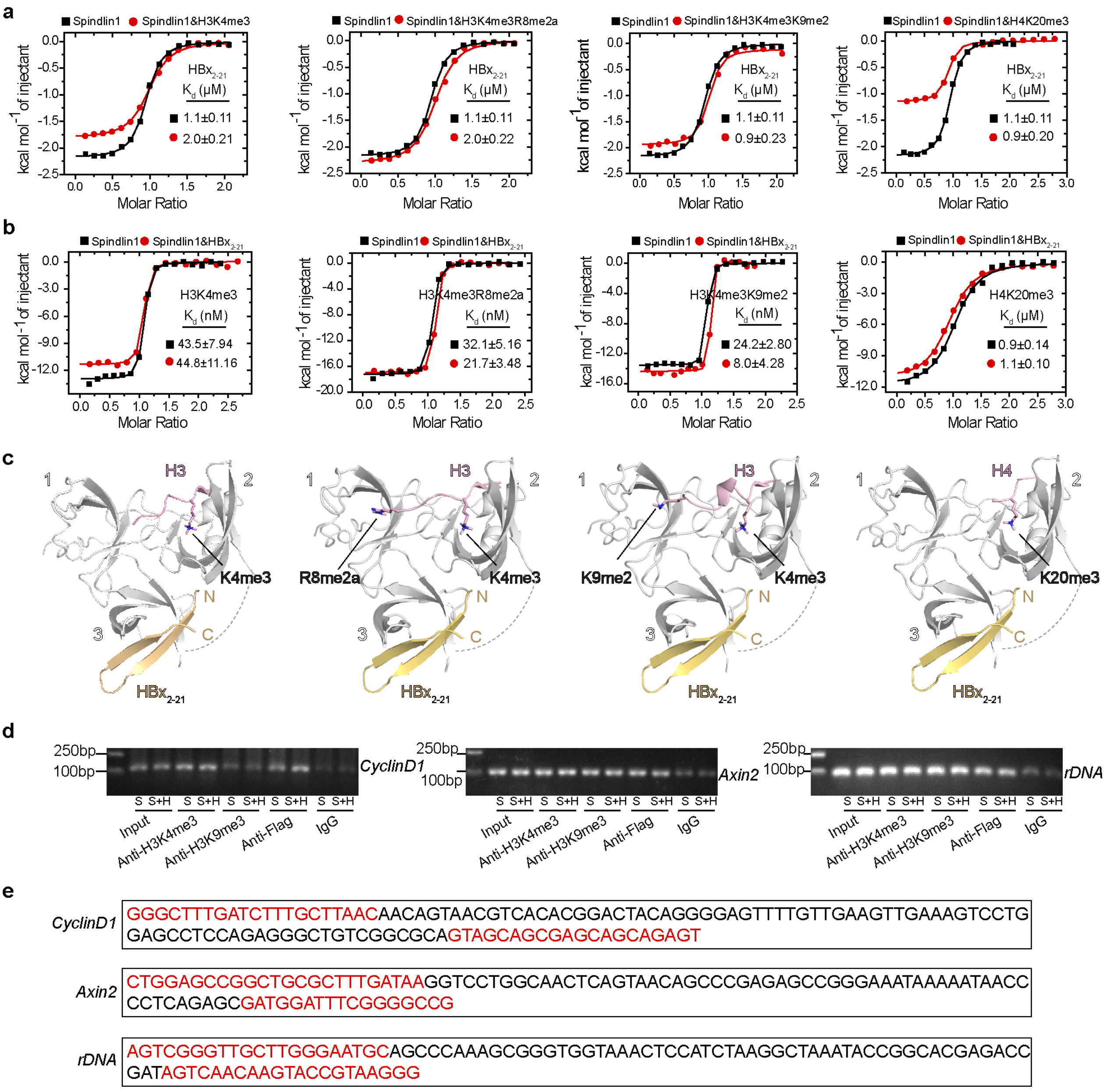


**Supplementary Figure 3. HBx engagement is compatible with histone binding by Spindlin1.** (a) ITC fitting curves of $HBx_{2\text{-}21}$ peptide titrated to free $Spindlin1_{50–262}$ protein or $Spindlin1_{50–262}$ protein incubated with H3K4me3, H3 “K4me3-R8me2a”, H3 “K4me3-K9me2” or H4K20me3 peptide. (b) ITC fitting curves of H3K4me3, H3 “K4me3-R8me2a”, H3 “K4me3-K9me2” or H4K20me3 peptide titrated to free $Spindlin1_{50–262}$ protein or $Spindlin1_{50–262}$ protein incubated with $HBx_{2\text{-}21}$ peptide. (c) Cartoon representation of structural comparison of Spindlin1-$HBx_{2\text{-}21}$ with Spindlin1-H3K4me3 (PDB ID 4MZG), Spindlin1-H3 “K4me3-R8me2a” (PDB ID 4MZF), Spindlin1-H3 “K4me3-K9me2” (PDB ID 7BU9) or Spindlin1-H4K20me3 (PDB ID 5Y5W). (d-e) DNA gel (d) and representative Sanger sequencing data (e) of ChIP-qPCR results in HEK 293 T cells, which were transfected with Flag-Spindlin1 (S) or Flag-Spindlin1 plus HBx (S+H). The bases marked in red represent the corresponding primer sequences used for qPCR analyses. n = 3 independent experiments.  Source data are provided as a Source Data file.

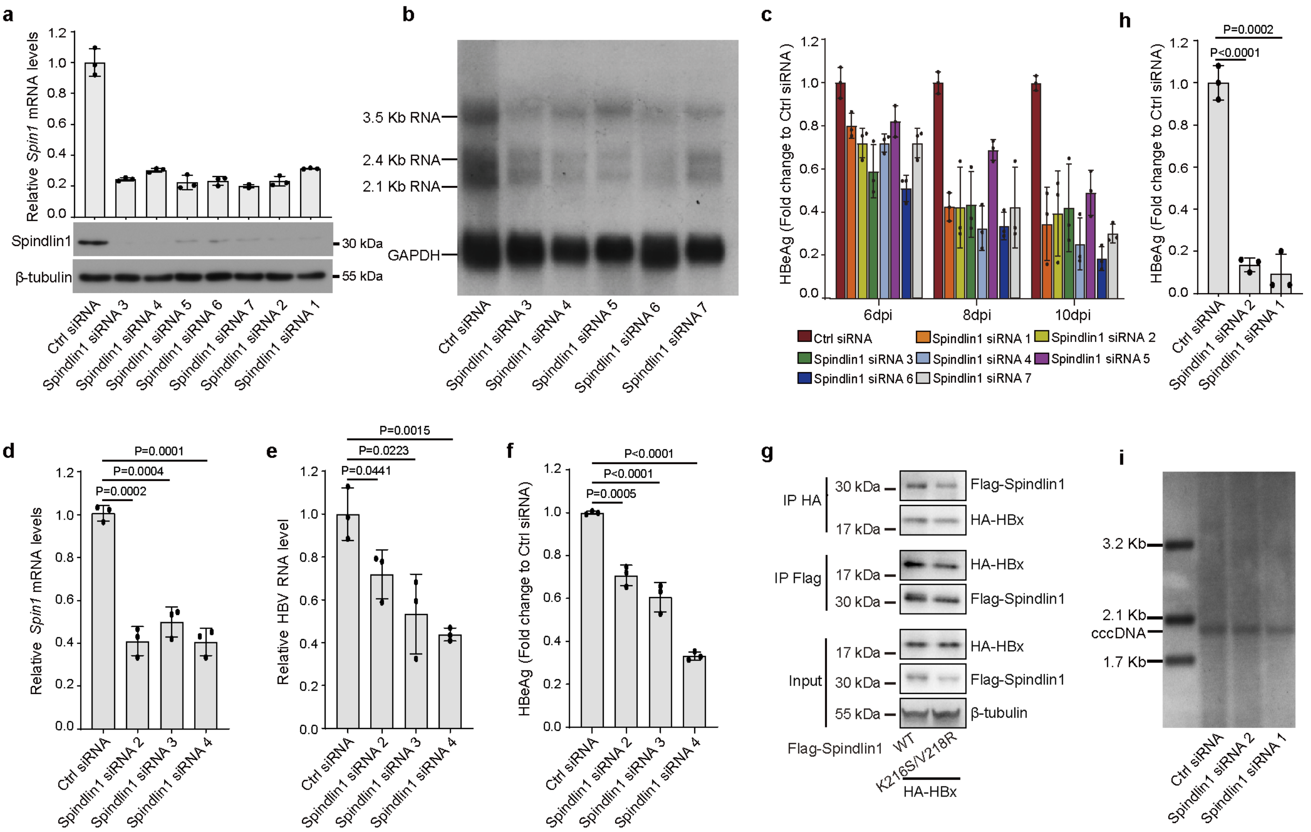


**Supplementary Figure 4. Spindlin1 is required for HBV transcription.** (a-c) We chose another five Spindlin1 siRNAs (3-7) to confirm the function of Spindlin1-HBx interaction in HBV transcription. (a) Detection of Spindlin1 knockdown efficiency in HBV infected HepG2-NTCP cells by WB and RT-qPCR analysis (Spindlin1 siRNAs versus Ctrl siRNA, *P*-values are 0.0001, 0.0002, 0.0002, 0.0001, 0.0001, 0.0001 and 0.0002, respectively from Spindlin1 siRNA 3 to 7, 2, 1). (b-c) (b) NB analysis of HBV transcription in HBV infected HepG2-NTCP cells, which were transfected with Ctrl siRNA or Spindlin1 siRNAs at 3dpi and collected at 10dpi for NB analysis. The corresponding culture medium were collected every two days for HBeAg analysis by ELISA (c). Spindlin1 siRNAs versus Ctrl siRNA, *P*-values are 0.0199, 0.0080, 0.0077, 0.0043, 0.0377, 0.0008 and 0.0080 at 6dpi, 0.0003, 0.0066, 0.0037, 0.0005, 0.0014, 0.0001 and 0.0066 at 8dpi, 0.0114, 0.0065, 0.0081, 0.0005, 0.0013, <0.0001 and <0.0001 at 10dpi, respectively from Spindlin1 siRNA 1 to 7. (d) Detection of Spindlin1 knockdown efficiency in HBV infected primary human hepatocytes (PHHs). (e-f) PHHs were transfected with Ctrl siRNA or Spindlin1 siRNAs (2, 3 and 4 tested here) for 16h and then infected with HBV. At 11dpi, cells and culture medium were harvest and HBV RNA levels and HBeAg levels were analyzed by RT-qPCR (e) and ELISA (f), respectively. (g) Co-IP assay assessing the interaction between HBx and Spindlin1 mutant in Huh7 cells. Cells were co-transfected with HA-tagged HBx and WT or K216S/V218R double mutant Flag-Spindlin1. After 48h, cellular extracts were immunoprecipitated with anti-Flag and anti-HA antibodies and analyzed using WB. β-tubulin was measured and analyzed as an input control. (h-i) HepG2-NTCP cells were transfected with Ctrl siRNA or Spindlin1 siRNAs for 48h and then infected with HBV virus. At 3dpi, culture medium and cells were harvested and HBeAg and HBV cccDNA levels were analyzed by ELISA (h) and Southern blot (i), respectively. (a, c-f, h) Data represent the mean±SD (n = 3 independent experiments). *P*-values between the groups were calculated with an unpaired two tailed *t* test. Source data are provided as a Source Data file.

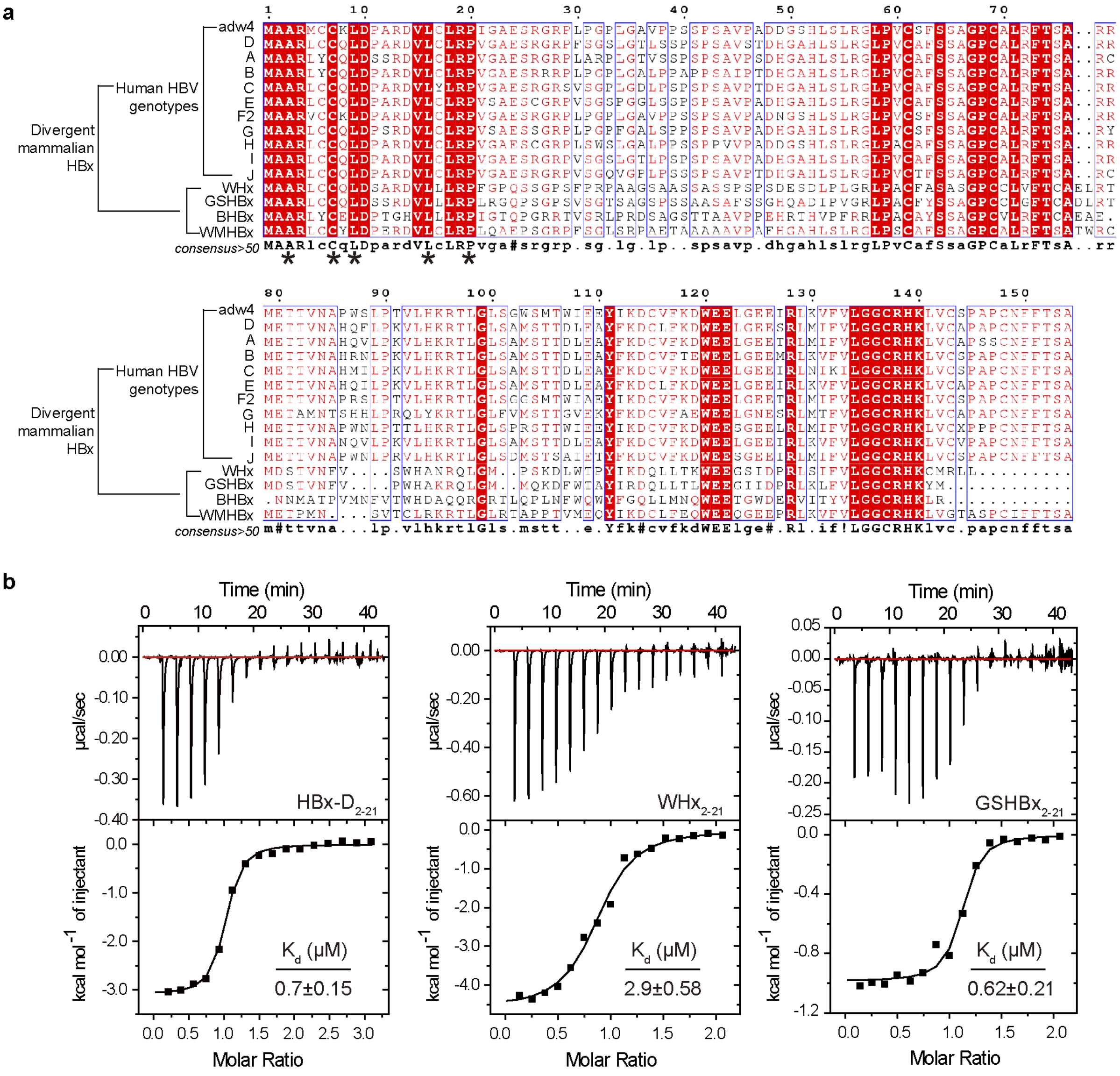


**Supplementary Figure 5. Conservation of Spindlin1-HBx interaction in mammals.** (a) Alignment of HBx protein sequences from various human HBV subtypes and mammals. * Key residues involved in Spindlin1 engagement. (b) ITC fitting curves of HBx-$D_{2\text{-}21}$, $WHx_{2\text{-}21}$ and $GSHBx_{2\text{-}21}$ peptides titrated to $Spindlin1_{50\text{–}262}$ protein. WHx: woodchuck HBx; GSHBx: ground squirrel HBx; BHBx: bat HBx; WMHBx: wooly monkey HBx.

**Supplementary Table 1. List of peptides used in this study.**

| Peptide name | Sequences |
|---|---|
| $HBx_{2-21}$ | AARMCCKLDPARDVLCLRPI-NH2 |
| $HBx_{11-30}$ | Ac-PARDVLCLRPIGAESRGRPL-NH2 |
| $HBx_{21-40}$ | Ac-IGAESRGRPLPGPLGAVPPS-NH2 |
| $HBx_{31-50}$ | Ac-PGPLGAVPPSSPSAVPADDG-NH2 |
| $HBx_{41-60}$ | Ac-SPSAVPADDGSHLSLRGLPV-NH2 |
| $HBx_{51-70}$ | Ac-SHLSLRGLPVCSFSSAGPCA-NH2 |
| $HBx_{61-80}$ | Ac-CSFSSAGPCALRFTSARRME-NH2 |
| $HBx_{71-90}$ | Ac-LRFTSARRMETTVNAPWSLP-NH2 |
| $HBx_{81-100}$ | Ac-TTVNAPWSLPTVLHKRTLGL-NH2 |
| $HBx_{91-110}$ | Ac-TVLHKRTLGLSGWSMTWIEE-NH2 |
| $HBx_{101-120}$ | Ac-SGWSMTWIEEYIKDCVFKDW-NH2 |
| $HBx_{111-130}$ | Ac-YIKDCVFKDWEELGEEIRLK-NH2 |
| $HBx_{121-140}$ | Ac-EELGEEIRLKVFVLGGCRHK-NH2 |
| $HBx_{131-154}$ | Ac-VFVLGGCRHKLVCSPAPCNFFTSA |
| $H3_{1–10}K4me3$ | ARTK(me3)QTARKS-NH2 |
| $H3_{1–20}K4me3$ | ARTK(me3)QTARKSTGGKAPRKQL-NH2 |
| $H3_{1–20}K4me3R8me2a$ | ARTK(me3)QTAR(me2a)KSTGGKAPRKQL-NH2 |
| $H3_{1–15}K4me3K9me2$ | ARTK(me3)QTARK(me2)STGGKA-NH2 |
| $H3_{1–15}K4me3K9me3$ | ARTK(me3)QTARK(me3)STGGKA |
| $H3_{1–20}K4me3K9me3$ | ARTK(me3)QTARK(me3)STGGKAPRKQL |
| $H4_{11–30}K20me3$ | Ac-GKGGAKRHRK(me3)VLRDNIQGIT-NH2 |
| $HBx\text{-}D_{2-21}$ | AARLCCQLDPARDVLCLRPV-NH2 |
| WHx | AARLCCQLDSARDVLLLRPF-NH2 |
| GSHBx | AARLCCQLDSSRDVLLLRPL-NH2 |

**Supplementary Table 2. Summary of thermodynamic parameters from isothermal titration calorimetry binding assays.**

| Protein | Peptide name | ΔH (kal/mol) | ΔS (cal/mol/deg) | K ($M^{-1}$) | N |
|---|---|---|---|---|---|
| Spindlin1$_{50\text{-}262}$ | HBx$_{2\text{-}21}$ | -2183±19.1 | 20 | 9.14E5±9.40E4 | 0.9±0.005 |
| | HBX$_{11\text{-}30}$ | -3496±197.8 | 8.2 | 2.26E4±4.32E3 | 1.2±0.029 |
| | HBx$_{61\text{-}80}$ | -6319±252.7 | -1.7 | 1.82E4±1.82E3 | 1±0.000 |
| | HBx-D$_{2\text{-}21}$ | -3096±42.71 | 18.0 | 1.56E6±2.43E5 | 0.951±0.009 |
| | FWHx | -4600±130.4 | 9.96 | 3.54E5±7.02E4 | 0.9±0.018 |
| | GSHBx | -987.3±23.4 | 25.3 | 1.83E6±6.32E5 | 1.1±0.017 |
| | HBx$_{2\text{-}21}$V15R | -854.0±51.42 | 19.4 | 7.36E4±1.78E4 | 1.0±0.049 |
| | HBx$_{2\text{-}21}$L18R | -840.2±102.4 | 17.7 | 3.11E4±9.95E3 | 1.1±0.083 |
| | HBx$_{2\text{-}21}$ V15E/C17R | N.D. | N.D. | N.D. | N.D. |
| | HBx$_{2\text{-}21}$AAAAA | N.D. | N.D. | N.D. | N.D. |
| | H3$_{1\text{-}20}$K4me3 | -12980±106.6 | -9.75 | 2.38E7±4.35E6 | 1.0±0.005 |
| | H3$_{1\text{-}20}$K4me3R8me2a | -17280±115.8 | -23.6 | 3.20E7±5.15E6 | 1.0±0.004 |
| | H3$_{1\text{-}15}$K4me3K9me2 | -14960±61.22 | -15.3 | 4.18E7±4.82E6 | 1.0±0.002 |
| | H3$_{1\text{-}20}$K4me3K9me3 | -13500±85.75 | -9.0 | 8.53E7±2.90E7 | 1.0±0.003 |
| | H4$_{11\text{-}30}$K20me3 | -12920±254.2 | -12.3 | 1.1E6±1.6E5 | 1.0±0.016 |
| Spindlin1$_{50\text{-}262}$ K216S/V218R | HBx$_{2\text{-}21}$ | N.D. | N.D. | N.D. | N.D. |
| Spindlin1$_{50\text{-}262}$ V232R | | N.D. | N.D. | N.D. | N.D. |
| Spindlin1$_{50\text{-}262}$ I245R | | N.D. | N.D. | N.D. | N.D. |
| Spindlin1$_{50\text{-}262}$&H3$_{1\text{-}20}$K4me3 | HBx$_{2\text{-}21}$ | -1803±19.54 | 20.1 | 5.07E5±5.28E4 | 1.0±0.007 |
| Spindlin1$_{50\text{-}262}$&H3$_{1\text{-}20}$K4me3R8me2a | | -2330±26.26 | 18.3 | 4.98E5±5.37E4 | 1.0±0.008 |
| Spindlin1$_{50\text{-}262}$&H3$_{1\text{-}15}$K4me3K9me2 | | -1913±34.77 | 21.5 | 1.24E6±3.23E5 | 1.0±0.016 |
| Spindlin1$_{50\text{-}262}$&H3$_{1\text{-}20}$K4me3K9me3 | | -1371±34.55 | 21.5 | 5.13E5±1.27E5 | 1.0±0.018 |
| Spindlin1$_{50\text{-}262}$&H4$_{11\text{-}30}$K20me3 | | -1145±17.93 | 23.9 | 1.17E6±2.58E5 | 0.8±0.009 |
| Spindlin1$_{50\text{-}262}$&HBx$_{2\text{-}21}$ | H3$_{1\text{-}20}$K4me3 | -11440±112.1 | -4.62 | 2.38E7±5.93E6 | 1.0±0.005 |
| | H3$_{1\text{-}20}$K4me3R8me2a | -17040±96.9 | -22.0 | 4.74E7±7.62E6 | 1.0±0.003 |
| | H3$_{1\text{-}15}$K4me3K9me2 | -14860±139.6 | -12.1 | 1.74E8±9.28E7 | 1.0±0.004 |
| | H4$_{11\text{-}30}$K20me3 | -15790±47.9 | -14.3 | 2.80E8±6.75E7 | 1.0±0.001 |
| | H4$_{11\text{-}30}$K20me3 | -11220±165.1 | -10.3 | 9.43E5±8.44E4 | 0.9±0.010 |

*N.D. not detectable*

**Supplementary Table 3. Primer sequences used in this study (for HBV primers the positions are indicated relative to EcoRI site).**

| Primer ID | Sequences | Experiment |
|---|---|---|
| β-actin_F | CGTCACCAACTGGGACGACA | RT-qPCR |
| β-actin_R | CTTCTCGCGGTTGGCCTTGG | RT-qPCR |
| Spindlin1_F | ACCCCATTCGGAAAGACACC | RT-qPCR |
| Spindlin1_R | CCATTCCCCTCTTTCCACCC | RT-qPCR |
| HBV_F | TCACCAGCACCATGCAAC | RT-qPCR |
| HBV_R | AAGCCACCCAAGGCACAG | RT-qPCR |
| Cyclin D1_F | CCGTCCATGCGGAAGATC | RT-qPCR |
| Cyclin D1_R | ATGGCCAGCGGGAAGAC | RT-qPCR |
| Axin2_F | AGTGTGAGGTCCACGGAAAC | RT-qPCR |
| Axin2_R | CTTCACACTGCGATGCATTT | RT-qPCR |
| Pre-rRNA_F | TGTCAGGCGTTCTCGTCTC | RT-qPCR |
| Pre-rRNA_R | AGCACGACGTCACCACATC | RT-qPCR |
| HBV cccDNA_F | GTGCACTTCGCTTCACCTCT (Positions 1579-1598) | ChIP-qPCR |
| HBV cccDNA_R | AGCTTGGAGGCTTGAACAGT (Positions 1859-1878) | ChIP-qPCR |
| rDNA_F | AGTCGGGTTGCTTGGGAATGC | ChIP-qPCR |
| rDNA_R | CCCTTACGGTACTTGTTGACT | ChIP-qPCR |
| Axin2_F | CTGGAGCCGGCTGCGCTTTGATAA | ChIP-qPCR |
| Axin2_R | CGGCCCCGAAATCCATCGCTCTGA | ChIP-qPCR |
| Cyclin D1_F | GGGCTTTGATCTTTGCTTAAC | ChIP-qPCR |
| Cyclin D1_R | ACTCTGCTGCTCGCTGCTAC | ChIP-qPCR |

**Supplementary Table 4. Sequences of siRNAs used in this study.**

| siRNA name | Sequences (5'-3') |
|---|---|
| Ctrl siRNA sense | UUCUCCGAACGUGUCACGUTT |
| Ctrl siRNA antisense | ACGUGACACGUUCGGAGAATT |
| Spindlin1 siRNA 1 sense | GCAAAGCAGUGGAACAUAUTT |
| Spindlin1 siRNA 1 antisense | AUAUGUUCCACUGCUUUGCTT |
| Spindlin1 siRNA 2 sense | GCAUUAUGCCUGAUUCCAATT |
| Spindlin1 siRNA 2 antisense | UUGGAAUCAGGCAUAAUGCTT |
| Spindlin1 siRNA 3 sense | GGAAUAUGCCAAAGAAGAUTT |
| Spindlin1 siRNA 3 antisense | AUCUUCUUUGGCAUAUUCCTT |
| Spindlin1 siRNA 4 sense | GCACCUGUCAUGAACACAUTT |
| Spindlin1 siRNA 4 antisense | AUGUGUUCAUGACAGGUGCTT |
| Spindlin1 siRNA 5 sense | GCACACUUGGCAGACACAATT |
| Spindlin1 siRNA 5 antisense | UUGUGUCUGCCAAGUGUGCTT |
| Spindlin1 siRNA 6 sense | GGACCAGGUGCCUGUAAAUTT |
| Spindlin1 siRNA 6 antisense | AUUUACAGGCACCUGGUCCTT |
| Spindlin1 siRNA 7 sense | CCCUGUUACCCAGUGGAAATT |
| Spindlin1 siRNA 7 antisense | UUUCCACUGGGUAACAGGGTT |
