# Supplementary material for: Molecular insights into Spindlin1-HBx interplay and its impact on HBV transcription from cccDNA minichromosome
Source: Nat Commun. 2023 Aug 3;14:4663. doi: 10.1038/s41467-023-40225-w (PMC10400593; doi:10.1038/s41467-023-40225-w)
Supplement: Supplementary file 3 — Reporting Summary [file 41467_2023_40225_MOESM3_ESM.pdf]

nature portfolio

Corresponding author(s): Haitao Li

Last updated by author(s): Jun 28, 2023

Reporting Summary

Nature Portfolio wishes to improve the reproducibility of the work that we publish. This form provides structure for consistency and transparency in reporting. For further information on Nature Portfolio policies, see our Editorial Policies and the Editorial Policy Checklist.

Statistics

For all statistical analyses, confirm that the following items are present in the figure legend, table legend, main text, or Methods section.

n/a	Confirmed	
[ ]	[x]	The exact sample size (*n*) for each experimental group/condition, given as a discrete number and unit of measurement
[ ]	[x]	A statement on whether measurements were taken from distinct samples or whether the same sample was measured repeatedly
[ ]	[x]	The statistical test(s) used AND whether they are one- or two-sided<br>*Only common tests should be described solely by name; describe more complex techniques in the Methods section.*
[x]	[ ]	A description of all covariates tested
[x]	[ ]	A description of any assumptions or corrections, such as tests of normality and adjustment for multiple comparisons
[ ]	[x]	A full description of the statistical parameters including central tendency (e.g. means) or other basic estimates (e.g. regression coefficient) AND variation (e.g. standard deviation) or associated estimates of uncertainty (e.g. confidence intervals)
[ ]	[x]	For null hypothesis testing, the test statistic (e.g. *F*, *t*, *r*) with confidence intervals, effect sizes, degrees of freedom and *P* value noted<br>*Give P values as exact values whenever suitable.*
[x]	[ ]	For Bayesian analysis, information on the choice of priors and Markov chain Monte Carlo settings
[x]	[ ]	For hierarchical and complex designs, identification of the appropriate level for tests and full reporting of outcomes
[x]	[ ]	Estimates of effect sizes (e.g. Cohen's *d*, Pearson's *r*), indicating how they were calculated

Our web collection on statistics for biologists contains articles on many of the points above.

Software and code

Policy information about availability of computer code

Data collection	Diffraction data were collected at beamline BL18U of the Shanghai Synchrotron Radiation Facility (SSRF)
Data analysis	1 SPRi data were calculated using Plexera Data Explorer Version-0.0.6956.16078.<br>2 TSA data were analyzed using Bio-Rad CFX-Manager 3.1.<br>3 ITC data were analyzed using MicroCal Origin 7.0.<br>4 Crystal data were processed and analyzed using HKL2000 v708, CCP4 7.0, Phenix Suite v1.15.2-3472, Coot v0.9.4.1 and Pymol 2.5.2.<br>5 The data from qPCR and ELISA assay were analyzed using GraphPad Prism 8.0.2 (263).

For manuscripts utilizing custom algorithms or software that are central to the research but not yet described in published literature, software must be made available to editors and reviewers. We strongly encourage code deposition in a community repository (e.g. GitHub). See the Nature Portfolio guidelines for submitting code & software for further information.

March 2021

Data

Policy information about availability of data

All manuscripts must include a data availability statement. This statement should provide the following information, where applicable:

- Accession codes, unique identifiers, or web links for publicly available datasets
- A description of any restrictions on data availability
- For clinical datasets or third party data, please ensure that the statement adheres to our policy

The atomic coordinates and structure factors for the reported complex structures of Spindlin1-HBx have been deposited in the Protein Data bank with accession number 8GTX [https://www.rcsb.org/structure /unreleased/8GTX]. The PDB codes of the previously determined structures used in this manuscript are: 4MZG [https://doi.org/10.2210/pdb4mzg/pdb] (Spindlin1-H3K4me3), 7E9M [https://doi.org/10.2210/pdb7 e9m/pdb] (Spindlin1-SPINDOC256-281), 7BQZ [https://doi.org/10.2210/pdb7bqz/pdb] (Spindlin1-H3"K4me3-K9me3"), 3I7H [https://doi.org/10.2210/pdb3I7H/pdb] (DDB1-HBx88-100), 5FCG [https://doi.org /10.2210/pdb5fcg/pdb] (Bcl-2-HBx110-135), 2NS2 [https://doi.org/10.2210/pdb2ns2/pdb] (Spindlin1), 4MZF [https://doi.org/10.2210/pdb4MZF/pdb] (Spindlin1-H3 "K4me3-R8me2a"), 7BU9 [https://doi.org/10.2210/ pdb7BU9/pdb] (Spindlin1-H3 "K4me3-K9me2"), 5Y5W [https://doi.org/10.2210/p db5Y5W/pdb] (Spindlin1-H4K20me3). The authors declare that all the data supporting the findings of this study are either shown in the main and supplementary text. Source data are provided with this paper.

Human research participants

Policy information about studies involving human research participants and Sex and Gender in Research.

Reporting on sex and gender	N/A
Population characteristics	N/A
Recruitment	N/A
Ethics oversight	N/A

Note that full information on the approval of the study protocol must also be provided in the manuscript.

Field-specific reporting

Please select the one below that is the best fit for your research. If you are not sure, read the appropriate sections before making your selection.

[x] Life sciences [ ] Behavioural & social sciences [ ] Ecological, evolutionary & environmental sciences

For a reference copy of the document with all sections, see nature.com/documents/nr-reporting-summary-flat.pdf

Life sciences study design

All studies must disclose on these points even when the disclosure is negative.

Sample size	No sample size calculation was performed. Independent biological repeats followed by statistical analysis were performed to confirm the results, sample sizes are reported in the figure legends.
Data exclusions	No data were excluded.
Replication	All experiments were performed at least two to three times and were reproducible.
Randomization	Randomization was not used since there are no experimental groups.
Blinding	No blinding strategy was employed in this study, as no subjective analysis was involved in any of the structural, biochemical and cell experiments.

Reporting for specific materials, systems and methods

We require information from authors about some types of materials, experimental systems and methods used in many studies. Here, indicate whether each material, system or method listed is relevant to your study. If you are not sure if a list item applies to your research, read the appropriate section before selecting a response.

Materials & experimental systems

n/a	Involved in the study
☐	☒ Antibodies
☐	☒ Eukaryotic cell lines
☒	☐ Palaeontology and archaeology
☒	☐ Animals and other organisms
☒	☐ Clinical data
☒	☐ Dual use research of concern

Methods

n/a	Involved in the study
☒	☐ ChIP-seq
☒	☐ Flow cytometry
☒	☐ MRI-based neuroimaging

Antibodies

Antibodies used

anti-Spindlin1 Rabbit mAb (Cell Signaling Technology, Cat.#: 89139, clone name: E6R1Z, Dilution: 1:1000 for immunoblotting and 1:50 for ChIP).
anti-DDB1 Rabbit mAb (Cell Signaling Technology, Cat.#: 6998, clone name: D4C8, Dilution: 1:1000 for immunoblotting).
anti-HA Rabbit mAb (Cell Signaling Technology, Cat.#: 3724, clone name: C29F4, Dilution: 1:2000 for immunoblotting and 1:50 for coimmunoprecipitation).
anti-H3K9me3 Rabbit pAb (Abcam, Cat.#: ab8898, Dilution: 1:50 for ChIP).
anti-Flag Mouse mAb (Sigma-Aldrich, Cat.#: F1804, clone name: M2, Dilution: 1:2000 for immunoblotting and 1:100 for ChIP).
anti-β-tubulin Mouse mAb (EarthOx, Cat.#: E021040, Dilution: 1:4000 for immunoblotting).
anti-H3K4me3 Rabbit mAb (Merck Millipore, Cat.#: 04-745, clone name: MC315, Dilution: 1:50 for ChIP).
anti-SMC6 Mouse mAb (abcepta, Cat.#: AT3956a, clone name: 2E7, Dilution: 1:1000 for immunoblotting).
Alexa Fluor 546 donkey anti-mouse IgG (Invitrogen, Cat.#: A10036, Dilution: 1:1000 for immunofluorescence).
anti-HBcAg antibody (1C10) was developed and validated by Prof. Wenhui Li's lab.

Validation

The antibodies (except HBcAg) employed in this research were commercially obtainable and were authenticated by the provider based on the information presented in the relevant data sheets.

anti-Spindlin1 Rabbit mAb (Cell Signaling Technology, Cat.#: 89139): https://www.cellsignal.com/products/primary-antibodies/spin1-e6r1z-rabbit-mab/89139
anti-DDB1 Rabbit mAb (Cell Signaling Technology, Cat.#: 6998): https://www.cellsignal.com/products/primary-antibodies/ddb-1-d4c8-rabbit-mab/6998
anti-HA Rabbit mAb (Cell Signaling Technology, Cat.#: 3724): https://www.cellsignal.com/products/primary-antibodies/ha-tag-c29f4-rabbit-mab/3724
anti-H3K9me3 Rabbit pAb (Abcam, Cat.#: ab8898): https://www.abcam.com/products/primary-antibodies/histone-h3-tri-methyl-k9-antibody-chip-grade-ab8898.html
anti-Flag Mouse mAb (Sigma-Aldrich, Cat.#: F1804): https://www.sigmaaldrich.cn/CN/en/product/sigma/f1804
anti-β-tubulin Mouse mAb (EarthOx, Cat.#: E021040): https://earthox.net/product/anti-beta-tubulin-mouse-monoclonal-antibody-50ul/
anti-H3K4me3 Rabbit mAb (Merck Millipore, Cat.#: 04-745): https://www.sigmaaldrich.cn/CN/en/product/mm/04745
anti-SMC6 Mouse mAb (abcepta, Cat.#: AT3956a): https://www.abcepta.com.cn/products/AT3956a-SMC6L1-Antibody-monoclonal-M01
Alexa Fluor 546 donkey anti-mouse IgG (Invitrogen, Cat.#: A10036): https://www.thermofisher.cn/cn/zh/antibody/product/Donkey-anti-Mouse-IgG-H-L-Highly-Cross-Adsorbed-Secondary-Antibody-Polyclonal/A10036

Anti-HBcAg antibody (1C10) was developed and validated by Prof. Wenhui Li's lab - Yan H, et al. Sodium taurocholate cotransporting polypeptide is a functional receptor for human hepatitis B and D virus[J]. elife, 2012, 1: e00049.

Eukaryotic cell lines

Policy information about cell lines and Sex and Gender in Research

Cell line source(s)

HEK 293T, ATCC, Cat# CRL-3216
HepG2, ATCC, Cat# HB8065
Huh-7, Cell Bank of Type Culture Collection, Chinese Academy of Sciences, Cat# 3111C0001CCC000679
PHHs, Shanghai RILD Inc, Cat# 00995
HepG2-NTCP stable cell line expressing human NTCP was generated from HepG2 cells

Authentication

HEK 293T, HepG2, Huh-7 and PHHs were authenticated by the provider.
HepG2-NTCP cell line was validated by PCR and WB, HBV and HDV infection.

Mycoplasma contamination

Cell lines were tested using MycoBlue Mycoplasma Detector and were negative.

Commonly misidentified lines
(See ICLAC register)

No commonly misidentified cell lines were used in the study.
